# An Autobioluminescent Method for Evaluating *In Vitro* and *In Vivo* Growth of Rhodococcus equi

**DOI:** 10.1128/spectrum.00758-22

**Published:** 2022-05-31

**Authors:** Yasunori Suzuki, Naho Sakaizawa, Shinji Takai, Hiroaki Kubota, Noeru Hasegawa, Yukako Sasaki, Tsutomu Kakuda

**Affiliations:** a Laboratory of Animal Hygiene, Kitasato University School of Veterinary Medicine, Aomori, Japan; b Department of Microbiology, Tokyo Metropolitan Institute of Public Health, Tokyo, Japan; Texas A&M University

**Keywords:** autobioluminescence, endogenous promoter, IVIS, *Rhodococcus equi*, RNA-Seq, virulence plasmid, whole-genome sequencing

## Abstract

A previously reported method for evaluating the intracellular growth of Rhodococcus equi using enhanced green fluorescent protein is unsuitable for the quantitative evaluation of the entire sample because the signal can be detected only in the excitation region. Therefore, we created an autobioluminescent *R. equi* using luciferase (*luxABCDE*). First, we connected *luxABCDE* to the functional promoter P*_aphII_* and introduced it into the chromosomes of ATCC33701 and ATCC33701_P-. Luminescence was detected in both transformants, and a correlation between the bacterial number and luminescence intensity in the logarithmic phase was observed, indicating that *luxABCDE* is functionally and quantitatively expressed in *R. equi*. The luminescence of ATCC33701 was significantly higher than that of ATCC33701_P- at 24 h after infection with J774A.1. Next, RNA-Seq analysis of ATCC33701 to search for endogenous high-expression promoters resulted in the upstream sequences of RS29370, RS41760, and *vapA* being selected as candidates. Luminescence was detected in each transformant expressing the *luxABCDE* using these upstream sequences. We examined the luminescence intensity by coexpressing the *frp* gene, an enhancer of the luciferase reaction, with *luxABCDE*. The luminescence intensity of the coexpressing transformant was significantly enhanced in J774A.1 compared with the non-coexpressing transformant. Finally, we examined the luminescence *in vivo*. The luminescence signals in the organs peaked on the third day following the administration of ATCC33701 derivatives in mice, but no luminescence signal was detected when the ATCC33701_P- derivative was administered. The autologous bioluminescent method described herein will enhance the *in vitro* and *in vivo* quantitative analysis of *R. equi* proliferation.

**IMPORTANCE** We established an autologous bioluminescent strain of *R. equi* and a method to evaluate its proliferation *in vitro* and *in vivo* quantitatively. This method overcomes the weakness of the fluorescence detection system that only measures the site of excitation light irradiation. It is expected to be used as an *in vitro* and *in vivo* growth evaluation method with excellent quantitative properties. In addition, it was suggested that the selection of a promoter that expresses *luxABCDE* could produce a luminescence with high intensity. Although this method needs further improvement, such as creating transformants that can maintain high luminescence intensity regardless of environmental changes such as temperature fluctuations, it is possible to observe bacterial growth over time in mice without killing them. Therefore, this method can be used to not only evaluate the pathogenicity of various wild and gene-deficient strains but also to screen preventive and therapeutic methods such as vaccines.

## INTRODUCTION

Rhodococcus equi is an aerobic, Gram-positive, facultative intracellular coccobacillus frequently isolated from soil and causes pyogenic pneumonia in foals aged 1 to 3 months (1). In addition, the bacterium is also isolated from purulent lesions of various animals, including pigs, cattle, goats, and humans (2–6). The pathogenicity of *R*. *equi* is attributed to its ability to survive within macrophages, a trait encoded by virulence-associated proteins (Vaps) on virulence plasmids (7). The major function of Vaps is generating a pH-neutral and hence growth-promoting intracellular niche by preventing the incorporation of the proton-pumping vacuolar ATPase complex into the membrane of phagosomes and its subsequent transfer to lysosomes (8).

The integration of reporter gene fusions into living cells has led to a wide range of bioreporter organisms that can specifically detect and quantify many chemical, biological, and physical agents (9). When a bioreporter interacts with its target, the response of the transcriptional reporter gene appears as an easily recognizable phenotypic signal, such as a colorimetric change, fluorescence, or luminescence, which can be measured and quantified through the interface of an appropriate analytical instrument. In a previous study, we created an enhanced green fluorescent protein (EGFP) expressing *R. equi* using the *Streptomyces* φC31 integrase-based integration vector pINT through the junction of the aminoglycoside 3′-phosphotransferase promoter (P*_aphII_*) and *egfp* (10). This method could be used to evaluate the growth of *R. equi* in cultured cells under a fluorescence microscope. However, fluorescence observation is not appropriate for wide-range observation that is necessary for quantitative measurement of the fluorescence intensity in a large number of bacterial cells because only a part of the observation field is irradiated with excitation light. Conversely, luminescence detection has no limit for the range of the observation field, and it provides a low background due to nonsimultaneous emission, resulting in a high signal-to-background ratio. Thus, luminescence detection will be more suitable for experiments that require high sensitivity and quantification.

Autobioluminescence does not require the addition of a substrate by expressing the bacterial luciferase (*lux*) genes, including factors necessary for luminescence (11). The five genes that constitute the operon are involved in this bioluminescence and were harbored by genera, such as *Vibrio*, *Photobacterium*, and *Xenorhabdus* in nature (12). Namely, the bacterial luciferase subunits are encoded by the *luxA* and *luxB*. In contrast, the fatty acid reductase complex, which performs the biosynthesis of aldehyde substrates for the luminescence reaction, is encoded by the *luxC*, *luxD*, and *luxE* (9, 12). In a series of luminescence reactions, reduced flavin mononucleotide (FMNH_2_) is oxidized to flavin mononucleotide (FMN), and a long-chain aliphatic aldehyde is oxidized to the corresponding carboxylic acid. Both products are recycled under consumption of cellular energy and this is performed by a flavin reductase (FRP) which reduces the oxidized FMN to FMNH_2_. It has also been reported that the addition of the FRP gene to assays for bacterial luciferases will result in continuous light emission due to the regeneration of FMNH_2_ and thus will eliminate the necessity for rapid mixing devices for injection of FMNH_2_ combined with the light detection equipment (11, 12). The *luxABCDE*-based bioluminescence system has been successfully employed to monitor the expression of certain genes and disease development in real-time in both Gram-negative and Gram-positive bacteria. For example, Contag et al. (13) showed in 1995 that bioluminescence could be used to monitor disease progression in living animals. These initial studies were conducted using a Gram-negative bacteria, Salmonella Typhimurium, and they demonstrated that the growth of bacteria and the effects of drugs could be monitored using this noninvasive technology. In 2000, Francis et al. (14) succeeded in expressing a modified *lux* operon, harbored by *Photorhabdus luminescence*, in Gram-positive bacteria. A shuttle vector cloned with this modified *lux* operon was transformed into Staphylococcus aureus and monitored using bioluminescence in living animals. However, the bioluminescent bacteria could only allow a short period (less than 48 h) *in vivo* because the plasmid was lost without antibiotic selection pressure. Hence, the following year, their group developed a method to create a transposon-based *lux* transposon cassette and incorporate it into the chromosomes of Gram-positive bacteria (15). The objective of this study was to develop a bioluminescent strain of *R. equi* that could be used to quantify its growth *in vitro* and *in vivo*, using the integrase-based integration vector pINT and promoters, to enhance the expression of *luxABCDE*.

## RESULTS

### Expression of Lux gene using *aphII* promoter and its luminescence intensity.

**FIG 1:**
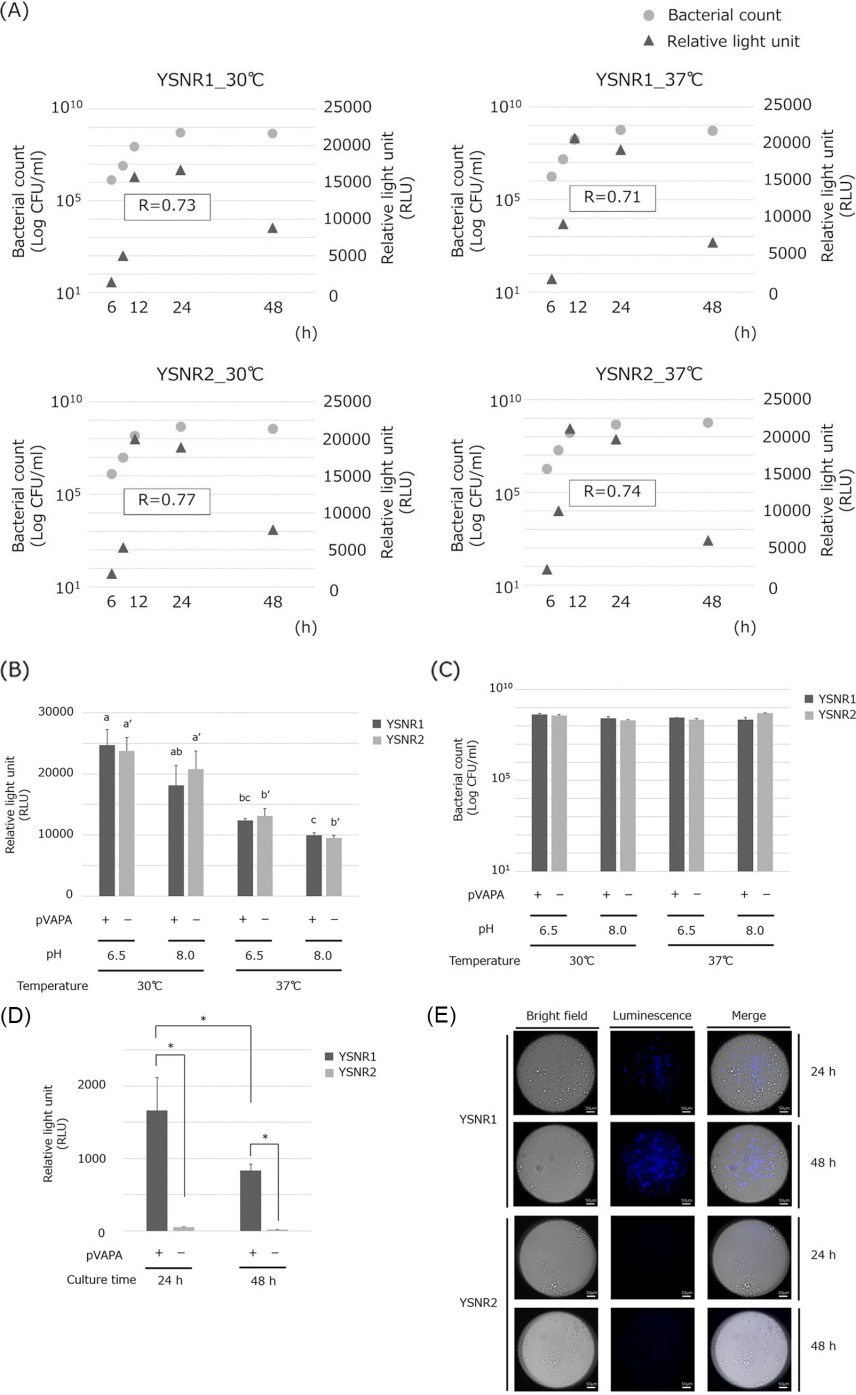
Luminescence of Rhodococcus equi transformants expressing the *lux* operon with the *aphII* promoter. (A) Relevance between bacterial growth and luminescence intensity. Each transformant (YSNR1 or YSNR2) was grown in BHI broth for any given time shown in the graphs at 30°C or 37°C with shaking (120 rpm). Experiments were performed in triplicate. The data are presented as the mean value and calculated the correlation coefficient between the two until 24 h of incubation. (B) Luminescence intensity and (C) actual bacteria count of bacterial solution after 24 h of incubation. Each transformant (YSNR1 or YSNR2) was grown in conditioned broth (20 g/L peptone, 8 g/L NaCl, and 300 mL/L horse meat extract) adjusted to pH 6.5 or 8.0 for 24 h at 30°C or 37°C with shaking (120 rpm). Experiments were performed in triplicate, and the data are presented as the mean value + standard deviation. Statistical analysis of changes in luminescence intensity in the same strains was performed and the data labeled with different letters YSNR1 without dash mark ('), YSNR2 with dash mark(') are significantly different from each other (*P *< 0.05). (D) Luminescence intensity when J774A.1 macrophage was infected with each transformant. Experiments were performed in triplicate, and the data are presented as the mean value + standard deviation. Data labeled connected by asterisks indicate significant differences (*P *< 0.05). (E) Intramacrophage growth of YSNR1 and YSNR2. J774A.1 macrophage was observed via luminescence microscope at 24- and 48-h postinfection. Bar = 50 μm.

The luminescence intensity of the culture broth of transformants YSNR1 (ATCC33701 transformed with pINT::P*_aphII_*-luxABCDE; see Tables 1 and 2 for details) and YSNR2 (ATCC33701_P- with pINT::P*_aphII_*-luxABCDE) was measured. We first examined the relationship between the number of bacteria and luminescence intensity of both transformants by collecting the culture solution at any given time and measuring the CFU/mL via the plating method and the luminescence intensity (relative light units; RLU) using a plate reader. In both strains, the correlation coefficients ranged within 0.71 to 0.77 from the logarithmic growth phase to the stationary phase (approximately 24 h), suggesting a positive correlation between bacterial growth and increase in luminescence intensity (Fig. 1A). At the time of inoculation of the precultured fungus solution (i.e., at 0 h of incubation) prepared using the brain–heart infusion (BHI) broth, luminescence could be detected in both YSNR1 (mean value ± standard deviation; 59.2 ± 7.7 RLU) and YSNR2 (56.0 ± 7.3 RLU) strains, and a significant difference was observed compared with BHI broth without inoculation. Therefore, the detection limit of the number of bacteria was set at 8.0 × 10^4^ CFU/mL (Table 3). In pH-conditioned media, 9,500 to 25,000 RLU was detected in all culture conditions and significantly higher luminescence intensity was observed at 30°C than at 37°C (Fig. 1B). There was no difference in the number of viable bacteria between YSNR1 and YSNR2 in each culture condition (Fig. 1C). J774A.1 cells were infected with the transformants. Initially, to confirm the intracellular viability of YSNR1 (carrying the virulence plasmid pVAPA) and YSNR2 (not carrying pVAPA) after infection, we collected the bacteria from the cells at certain intervals (1, 2, 4, 8, and 24 h) after infection. As shown in Fig. S1, the number of YSNR2 colonies at the 8 h was significantly lower than that of YSNR1. Additionally, the number of YSNR2 colonies decreased significantly with time, indicating that the bacteria did not grow and died. Next, luminescence intensity was measured; YSNR1 showed luminescence at 24 and 48 h after infection. However, the luminescence intensity decreased in 48 h of incubation compared with 24 h of incubation. On the other hand, the YSNR2 did not show luminescence (Fig. 1D). The J774A.1 cells were infected with each transformant, and the intracellular luminescence was observed using a luminescence microscope. Consistent with the results measured by the plate reader, the strong signal of intracellular luminescence was observed at 24-h postinfection in the YSNR1, but no luminescence was observed in the YSNR2 (Fig. 1E).

**TABLE 1:** Bacterial strains used in this study

Strain names	Description	Reference
Rhodococcus equi		
ATCC33701	A strain harboring virulence plasmid, pVAPA, isolated from a foal with pyogenic pneumonia	26
ATCC33701_P-	Artificially pVAPA-cured ATCC33701	26
YSNR1	ATCC33701 transformed with pYSN1	This study
YSNR2	ATCC33701_P- transformed with pYSN1	This study
YSNR3	ATCC33701 transformed with pYSN2	This study
YSNR4	ATCC33701 transformed with pYSN3	This study
YSNR5	ATCC33701 transformed with pYSN4	This study
YSNR6	ATCC33701_P- transformed with pYSN2	This study
YSNR7	ATCC33701_P- transformed with pYSN3	This study
YSNR8	ATCC33701_P- transformed with pYSN4	This study
YSNR9	ATCC33701 transformed with pYSN5	This study
YSNR10	ATCC33701 transformed with pYSN6	This study
YSNR11	ATCC33701_P- transformed with pYSN5	This study
YSNR12	ATCC33701_P- transformed with pYSN6	This study
Escherichia coli		
HST08	Commercially available competent cells (Genotype: F−, endA1, supE44, thi-1, recA1, relA1, gyrA96, phoA, Φ80dlacZΔM15, Δ(lacZYA-argF)U169, Δ(mrr-hsdRMS-mcrBC), ΔmcrA, λ−)	Takara bio
Vibrio parahaemolyticus		
NAHA-1	Environmental isolate	This study

**TABLE 2:** Plasmids used in this study

Plasmid names	Description	Reference
pINT	pUC57::aac(3)IV-integrase	23
pINT::P*_aphII_*-egfp	Vector for the integration of *egfp* using pINT	10
pMV306G13+Lux	pMV306hsp+LuxABCDE derivative in which P*_hsp60_* has been replaced with P*_G13_*	27
pYSN1	pINT::P*_aphII_*-luxABCDE	This study
pYSN2	pINT::P*_29370_*-luxABCDE	This study
pYSN3	pINT::P*_41760_*-luxABCDE	This study
pYSN4	pINT::P*_vapA_*-luxABCDE	This study
pYSN5	pINT::P*_aphII_*-luxABCDE-P*_aphII_*-frp	This study
pYSN6	pINT::P*_29370_*-luxABCDE-P*_29370_*-frp	This study

**TABLE 3:** Measurement of the detection limits of the autobioluminescence system^*a*^

Strain	Measurement item	0 h^*b*^	1 h	2 h	3 h
YSNR1	RLU^*c*^	59.2 ± 7.7^b^	94.0 ± 12.9^c^	173.2 ± 21.7^d^	302.0 ± 31.0^e^
	Bacterial count (CFU/mL)	8.6 × 10^4^ ± 1.3 × 10^4 A^	9.7 × 10^4^ ± 1.6 × 10^4 A,B^	1.3 × 10^5^ ± 9.5 × 10^3 B^	3.1 × 10^5^ ± 2.5 × 10^4 C^
YSNR2	RLU	56.0 ± 7.3^b'^	108.8 ± 8.1^c'^	186.6 ± 31.3^d'^	285.4 ± 21.4^e'^
	Bacterial count (CFU/mL)	8.1 × 10^4^ ± 1.2 × 10^4 A’^	1.0 × 10^5^ ± 2.0 × 10^4 A’^	1.4 × 10^5^ ± 8.3 × 10^3 A’^	3.8 × 10^5^ ± 8.6 × 10^4 B’^
BHI	RLU	5.4 ± 2.9^a,a'^			
	Bacterial count (CFU/mL)	0			

a Experiments were performed in triplicate, and the data are presented as the mean value ± standard deviation. Tests for significant differences were performed among numbers labeled with uppercase letters (A, B, C,…), among numbers labeled with uppercase letters with commas (A’, B’, C’,…), among numbers labeled with lowercase letters (a, b, c,…) or among numbers labeled with lowercase letters with commas (a’, b’, c’,…), respectively, and data labeled with different letters are significantly different from each other (*P* < 0.05).

b 0 h, immediately after inoculation.

c RLU, relative light units.

### Prediction of endogenous high-expression promoter of *R. equi* by RNA-Seq.

**FIG 2:**
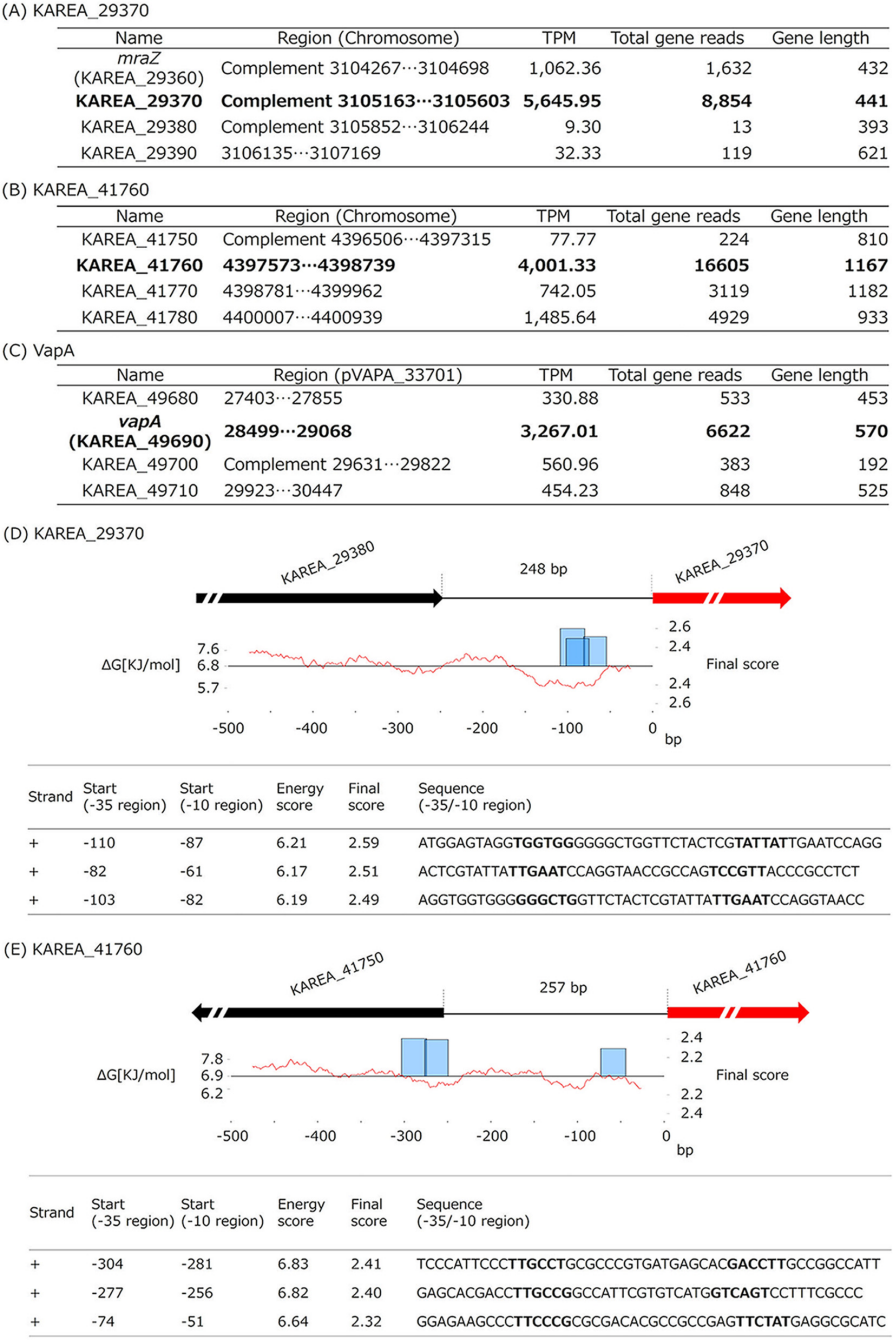
Comprehensive gene expression analysis and prediction of endogenous high-expression promoters of ATCC33701. Quantification of the expression levels of genes around KAREA_29370 (A), KAREA_41760 (B), and VapA (C). TPM is a value that indicates how many transcripts are present per million total transcripts in a sample, which is a practical method to quantify the amount of mRNA. Search for −10/−35 sequences within the upstream sequence of KAREA_29370 (D) and KAREA_41760 (E). The −10/−35 consensus sequence of *Streptomyces* sp. (16) was used as a matrix for the search. The boldface in the sequence indicates predicted −10/−35 sequences.

To search for promoter sequences that are not affected by temperature, considering the application to infection experiments *in vivo*, comprehensive gene expression analysis was performed using RNA extracted from ATCC33701 cultured under 37°C, pH 6.5 conditions. All RNA-Seq reads were mapped to the ATCC33701 chromosome (AP025268) and pVAPA plasmid (AP025269) determined in this study (an overview of the ATCC33701 genome is given in Table 4). Transcripts per million (TPM) values of all the genes in ATCC33701 were calculated, and the regions where the TPM values varied more than 10-fold between neighboring genes were extracted. Looking at the whole gene expression, we focused on the regions around the following three genes. (i) TPM value of the gene locus tag: KAREA_29370 (DUF3040 domain-containing protein; putative membrane protein) was 5,645.95, and the TPM values of the upstream gene (Locus tag: KAREA_29380) was 9.30. (ii) TPM value of the gene locus tag: KAREA_41760 (3-ketosteroid-9-alpha-hydroxylase subunit A) was 4001.33, and the TPM values of the upstream gene (Locus tag: KAREA_41750) were 77.77. (iii) TPM value of the VapA gene (KAREA_49690) was 3,267.01, and the TPM values of the upstream gene (KAREA_49680) was 330.48 (Fig. 2A, B, and C). Next, we searched for the −10/−35 consensus sequence reported in *Streptomyces* sp. (16) in the upstream sequences of the KAREA_29370 and KAREA_41760 genes. The candidate −10/−35 region was found around 150 bp upstream of the KAREA_29370, and the candidate −10/−35 region was found at 300 bp upstream and 150 upstream of the KAREA_41760 (Fig. 2D and E). Conversely, the upstream sequence of the VapA gene has been analyzed, and the transcription start site and −10/−35 region have been reported (17, 18). We predicted these regions as endogenous high-expression promoters and designated 500 bp upstream of the KAREA_29370 gene as P*_29370_*, 500 bp upstream of the KAREA_41760 gene as P*_41760_*, and 692 bp upstream of VapA gene as P*_vapA_*, respectively, for the following experiments wherein we bound these promoter sequences to *luxABCDE* and compared the changes in luminescence under different culture conditions.

**TABLE 4:** Assembly stats, general genome information, and relevant characteristics of Rhodococcus equi ATCC33701 strain

Strain and genome information	Data for Rhodococcus equi ATCC33701 strain:
Assembly and genome stats	
MinION	
No. of reads	165,169
Total no. of bases	1,407,030,597
Trimmed with NanoFilt	
No. of reads	123,939
Read length N50	13,882
Total no. of bases	1,076,229,772
MiSeq	
No. of reads	570,300
Total no. of bases	126,999,761
Trimmed with Trim Galore	
No. of reads	570,300
Total no. of bases	114,183,101
Coverage (×)	224.2
Chromosome description^*a*^	
Genome size (bp)	5,227,764
G+C content (%)	68.7
No. of CDSs^*b*^	4,915
Coding ratio (%)	91.3
No. of rRNA	15
No. of tRNA	63
No. of CRISPRS	0
pVAPA description^*a*^	
Genome size (bp)	80,602
G+C content (%)	64.6
No. of CDSs^*b*^	76
Coding ratio (%)	71.5
No. of rRNA	0
No. of tRNA	0
No. of CRISPRS	0

a All genomic stats are output from DFAST pipeline.

b CDSs, coding sequences.

### Expression of Lux gene using endogenous promoter and its luminescence intensity.

**FIG 3:**
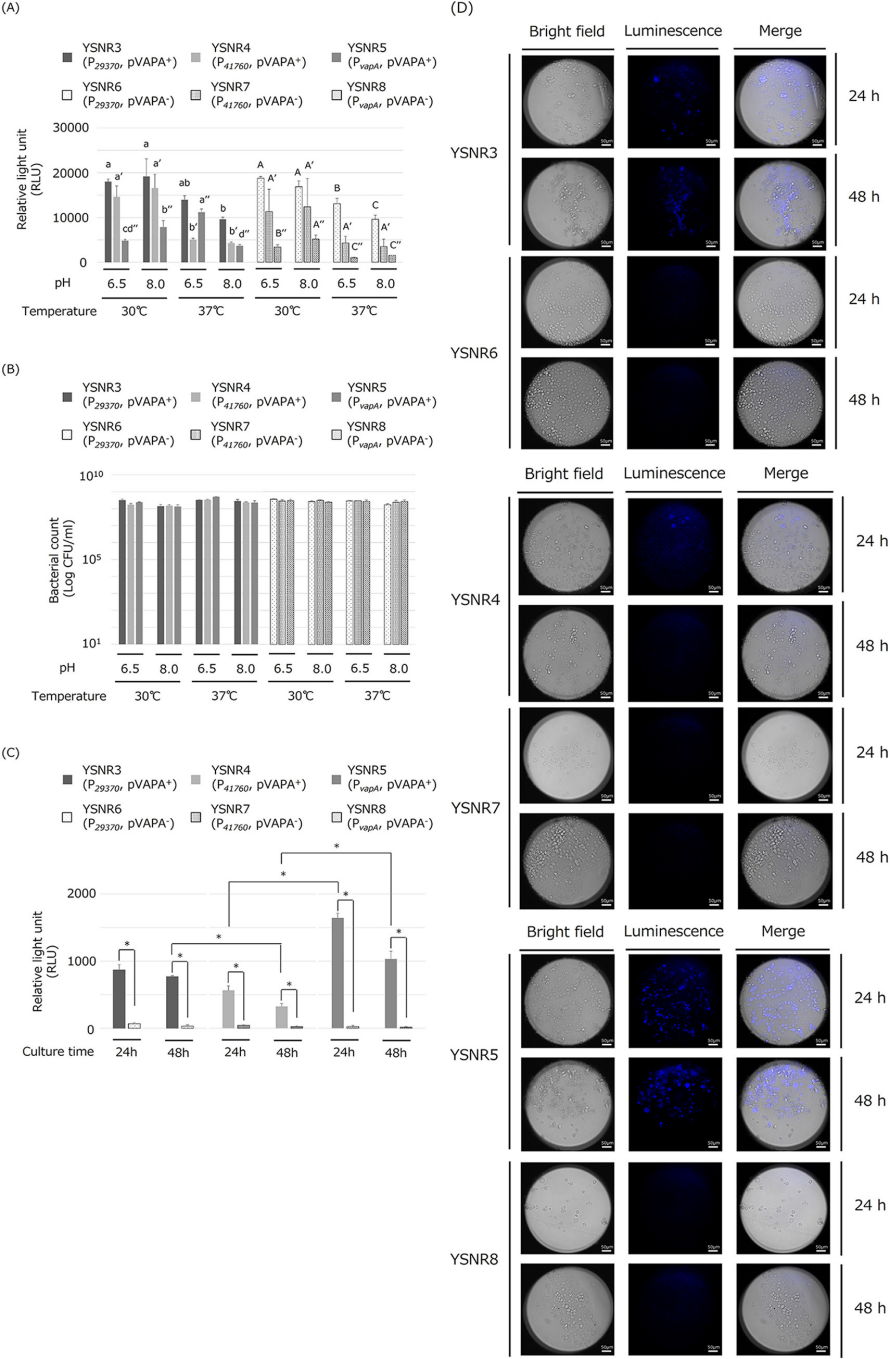
Luminescence of Rhodococcus equi transformants expressing the *lux* operon with the endogenous promoter (A) Luminescence intensity and (B) actual bacteria count of bacterial solution after 24 h of incubation. Each transformant (YSNR3, YSNR4, YSNR5, YSNR6, YSNR7, or YSNR8) was grown in conditioned broth adjusted to pH 6.5 or 8.0 for 24 h at 30°C or 37°C with shaking (120 rpm). Experiments were performed in triplicate, and the data are presented as the mean value + standard deviation. Statistical analysis of changes in luminescence intensity in the same strains was performed and the data labeled with different letters YSNR3 to 5 are lowercase, YSNR6 to 8 are uppercase. YSNR4 and YSNR7 with single dash mark ('), YSNR5 and YSNR8 with double dash mark ('') are significantly different from each other (*P *< 0.05). (C) Luminescence intensity when J774A.1 macrophage was infected with each transformant. Experiments were performed in triplicate, and the data are presented as the mean value + standard deviation. Data labeled connected by asterisks indicate significant differences (*P *< 0.05). (D) Intramacrophage growth of YSNR3, YSNR4, YSNR5, YSNR6, YSNR7, and YSNR8. J774A.1 macrophage was observed via luminescence microscope at 24- and 48-h postinfection. Bar = 50 μm.

The luminescence intensities of the culture broth of transformants YSNR3 (ATCC33701 transformed with pINT::P*_29370_*-luxABCDE; see Tables 1 and 2 for details), YSNR4 (ATCC33701 transformed with pINT::P*_41760_*-luxABCDE), YSNR5 (ATCC33701 transformed with pINT::P*_vapA_*-luxABCDE), YSNR6 (ATCC33701_P- transformed with pINT::P*_29370_*-luxABCDE), YSNR7 (ATCC33701_P- transformed with pINT::P*_41760_*-luxABCDE), and YSNR8 (ATCC33701_P- transformed with pINT::P*_vapA_*-luxABCDE) were measured. The YSNR3 and YSNR6, expressing the Lux gene using P*_29370_*, showed the highest luminescence intensity, exceeding 9,000 RLU in all culture conditions (Fig. 3A). Significantly higher luminescence intensity was observed in the 30°C incubation than in the 37°C incubation, except for the YSNR5. In contrast, the YSNR5 had the highest luminescence intensity when cultured at 37°C, pH 6.5 (Fig. 3A). There was no difference in the number of viable bacteria among YSNR3, YSNR4, YSNR5, YSNR6, YSNR7, and YSNR8 in each culture condition (Fig. 3B). J774A.1 cells were infected with these transformants, and the luminescence intensity was measured. The YSNR3, YSNR4, and YSNR5, carrying the virulence plasmid pVAPA, showed luminescence at 24 and 48 h after infection, whereas the YSNR6, YSNR7, and YSNR8, not carrying pVAPA, did not show luminescence. The luminescence intensity decreased 48-h postinfection. YSNR5 showed the highest luminescence intensity in J774A.1 cells, followed by YSNR3 and YSNR4 (Fig. 3C). J774A.1 cells were infected with each transformant, and the intracellular luminescence was observed using a luminescence microscope. Consistent with the results measured by the plate reader, the strong signal of intracellular luminescence was observed 24 h postinfection in the YSNR5, followed by YSNR3 and YSNR4. However, no luminescence signal was observed in the YSNR6, YSNR7, and YSNR8 (Fig. 3D).

### Change in luminescence intensity by addition of *frp* gene.

**FIG 4:**
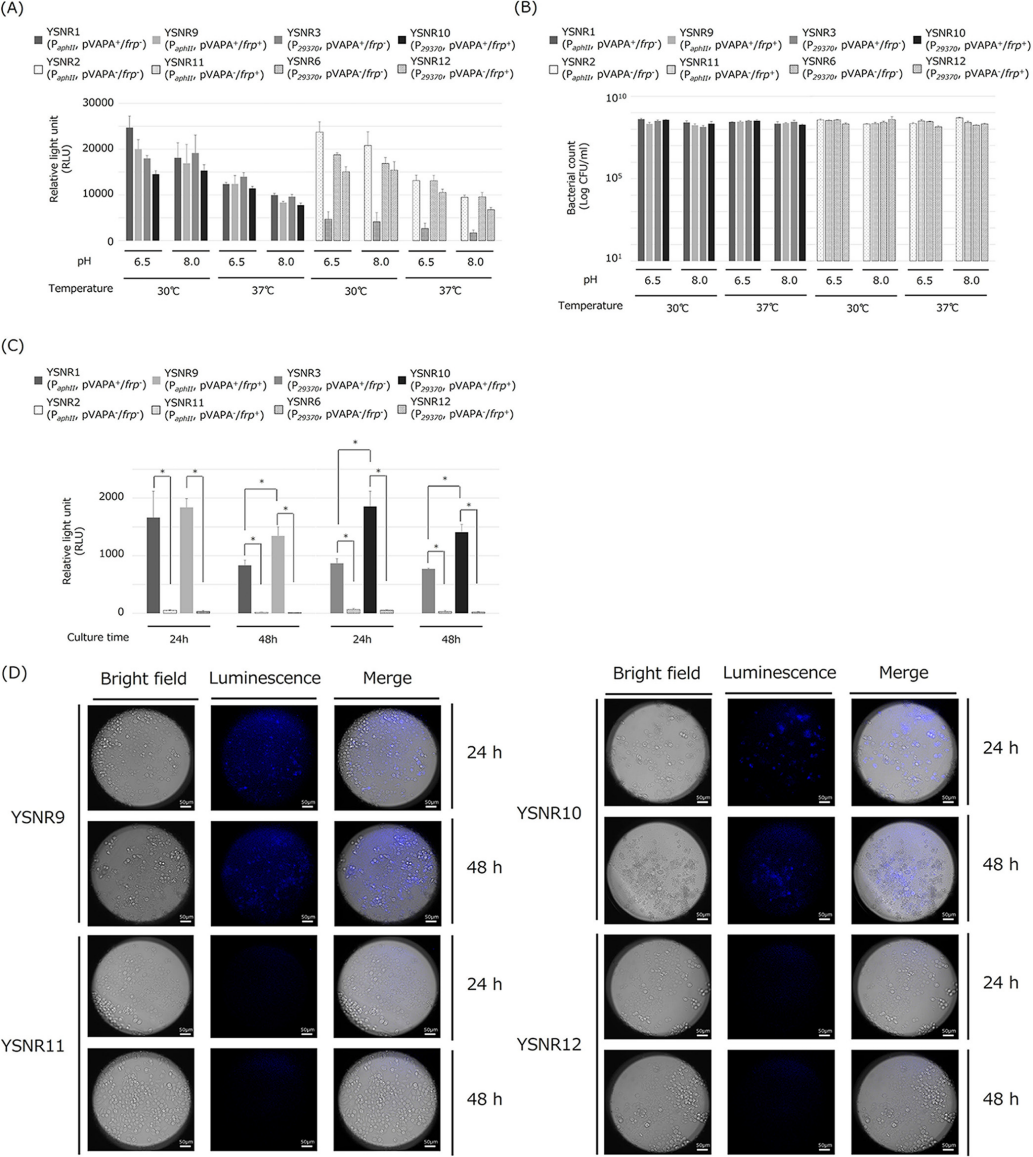
Change in luminescence intensity by addition of *frp* gene. (A) Luminescence intensity and (B) actual bacteria count of bacterial solution after 24 h of incubation. Each transformant (YSNR9, YSNR10, YSNR11, or YSNR12) was grown in conditioned broth adjusted to pH 6.5 or 8.0 for 24 h at 30°C or 37°C with shaking (120 rpm). Experiments were performed in triplicate and the data are presented as the mean value + standard deviation. The data of YSNR1, YSNR2, YSNR3, and YSNR6 are the same as shown in Fig. 1B and are depicted in the graph as a comparator strain without the *frp* gene. (C) Luminescence intensity when J774A.1 macrophage were infected with each transformant. Experiments were performed in triplicate, and the data are presented as the mean value + standard deviation. The data of YSNR1, YSNR2, YSNR3, and YSNR6 are the same as shown in Fig. 1D and are depicted in the graph as a comparator strain without the *frp* gene. Data labeled connected by asterisks indicate significant differences (*P *< 0.05). (D) Intramacrophage growth of YSNR9, YSNR10, YSNR11, and YSNR12. J774A.1 macrophage was observed via luminescence microscope at 24- and 48-h postinfection. Bar = 50 μm.

The luminescence intensities of the culture broth of transformants YSNR9 (ATCC33701 transformed with pINT::P*_aphII_*-luxABCDE-P*_aphII_*-frp; see Tables 1 and 2 for details), YSNR10 (ATCC33701 transformed with pINT::P*_29370_*-luxABCDE-P*_29370_*-frp), YSNR11 (ATCC33701_P- transformed with pINT::P*_aphII_*-luxABCDE-P*_aphII_*-frp), and YSNR12 (ATCC33701_P- transformed with pINT::P*_29370_*-luxABCDE-P*_29370_*-frp), to which the *frp* gene was added, were compared with those of YSNR1, YSNR2, YSNR3, and YSNR6, respectively. No significant change in the luminescence intensity was observed between the *frp*-integrated and nonintegrated transformants when either promoter (P*_aphII_* or P*_29370_*) was used to express, and the intensity tended to be the same or decreased in the *frp*-integrated transformants (Fig. 4A). In addition, there was no difference in the number of viable bacteria among YSNR1, YSNR2, YSNR3, YSNR6, YSNR9, YSNR10, YSNR11, and YSNR12 in each culture condition (Fig. 4B). J774A.1 cells were infected with these transformants, and the luminescence intensity was measured. The YSNR9 and YSNR10, carrying the virulence plasmid pVAPA, showed luminescence at 24 h postinfection. The luminescence intensity decreased in the 48-h culture, whereas the YSNR11 and YSNR12, not carrying pVAPA, did not show luminescence. In addition, the *frp*-integrated transformants showed significantly higher luminescence values than the nonintegrated strains (Fig. 4C). J774A.1 cells were infected with these transformants, and the intracellular growth and luminescence of the bacteria were observed using a luminescence microscope. Consistent with the results measured by the plate reader, the strong signal of intracellular luminescence was observed 24-h postinfection in the YSNR9 and YSNR10. However, no luminescence signal was observed in the YSNR11 and YSNR12 (Fig. 4D).

### Detection of an autobioluminescent Rhodococcus equi proliferating in the liver and spleen of the mice.

**FIG 5:**
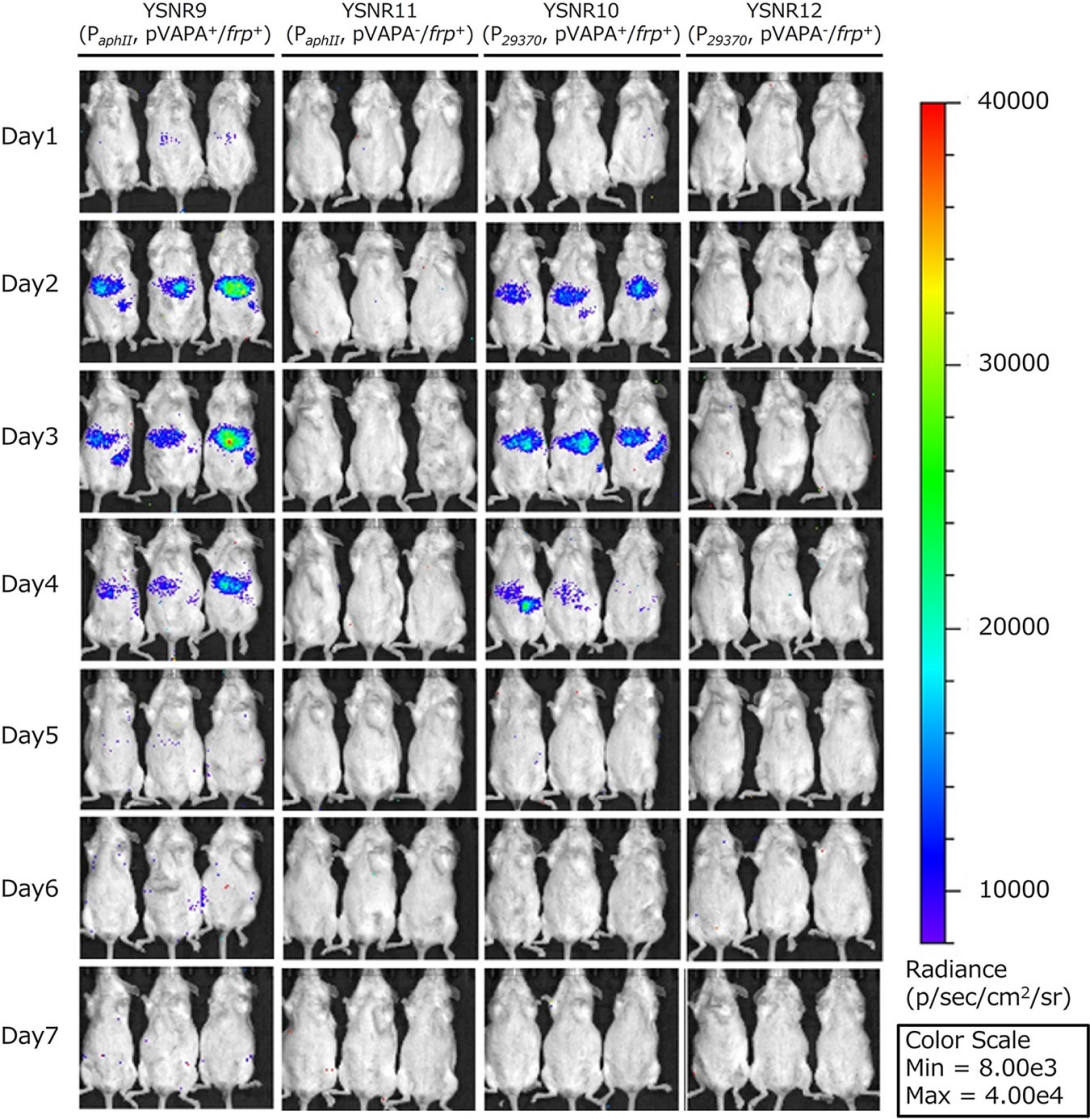
Growth of pVAPA-harboring and pVAPA-cured transformants in mouse liver and spleen. The luminescence signals of YSNR9, YSNR10, YSNR11, and YSNR12 were detected by *in vivo* imaging system (IVIS) during the 1-day time course.

*In vivo* imaging system (IVIS) was used to evaluate the proliferative potential of each transformant in the abdominal organs of the mice. Luminescence signals were observed in the region of the liver and spleen of mice inoculated with YSNR9 and YSNR11 after 1 day. Moreover, the area and intensity of luminescence increased over time until 3 days after administration and decreased thereafter. Nonetheless, the luminescence signals of pVAPA-cured transformants, including YSNR10 and YSNR12, were not detected at any observation date (Fig. 5). These results indicating an increase and decrease were consistent with previous reports wherein the wild-type strains, ATCC33701, and ATCC33701_*P*^−^ were administered, following which the numbers of bacteria in the liver and spleen were measured every few days (19).

## DISCUSSION

Our laboratory and others have established a semiquantitative method for detecting EGFP-expressing *R. equi* under a fluorescence microscope to evaluate the intracellular growth and pathogenicity in cultured cells (10, 20). The fluorescence detection system using green fluorescent protein (GFP) is an effective method for qualitative localization because only a part of the sample under test is irradiated with excitation light. However, it is difficult to evaluate the fluorescence intensity of the entire sample quantitatively. Conversely, the luminescence-based detection system is suitable for experiments to detect high sensitivity and quantitative signals because there is usually no spontaneous emission in the samples, and a higher S/B ratio can be obtained. The luminescence system has also been applied to some bacteria. It is a popular method for quantitative evaluation of infection and growth both *in vivo* and *in vitro* (9, 13–15). Still, there have been few reports on the application of this system to *R. equi* (21). This study aimed to establish a highly sensitive and quantitative method to evaluate the pathogenicity of *R. equi* using an autologous bioluminescence system.

The aminoglycoside phosphotransferase gene (*aphII*) is a kanamycin- and neomycin-resistance gene (22). We have previously reported expressing the EGFP gene using the promoter of this gene (P*_aphII_*) and gene complementation (10). In this study, we first generated transformants (YSNR1 and YSNR2) where the *luxABCDE* operon sequence was linked directly under P*_aphII_* in the integrase-based integration vector pINT (23) and examined whether luminescence signals could be detected. As shown in Fig. 1A, there is a positive correlation between the incubation period (i.e., growth of the bacteria) and the increase in luminescence intensity, indicating that the *luxABCDE* operon is functionally expressed in *R. equi* and can be used as an autologous bioluminescence system. This system can detect a significant difference in the number of bacteria of approximately 8.0 × 10^4^ CFU/mL immediately after inoculation and a slight increase in the number of bacteria in the early stage of logarithmic growth (Table 3), although it might be ineffective for experiments longer than 24 h, indicating it to be a highly quantitative and sensitive assay. Interestingly, the luminescence intensity tended to be higher at 30°C than at 37°C in pH-conditioned broth, even though there was no remarkable difference in the growth rate of both transformants (Fig. 1B and C). This suggests that P*_aphII_* may be a temperature-sensitive promoter activated under low-temperature conditions. Because *Streptomyces* and *Rhodococcus* are soil environmental bacteria, it is expected that their normal habitat temperature range is closer to 30°C, and this promoter activity may be higher.

We verified the proliferation of transformants in the cultured macrophages. We found that the YSNR1, ATCC33701-derived transformant showed stronger luminescence signals than the YSNR2, ATCC33701_P–derived transformant 24-h postinfection, and the signals by the YSNR1 decreased at 48 h (Fig. 1D). Similar results were also obtained in observations under a luminescence microscope (Fig. 1E). This result is consistent with the semiquantitative detection performed previously using GFP-expressed ATCC33701 and ATCC33701_P- strains (10). In preliminary experiments with or without adding amikacin to Dulbecco’s Modified Eagle Medium (DMEM), the luminescence intensity was significantly higher as the amikacin concentration decreased after 4 h of incubation in both YSNR1 and YSNR2 (Fig. S2). In addition, the luminescence intensity of YSNR1 and YSNR2 was 19,417 ± 2,267 (presented as the mean value ± standard deviation) RLU and 17,102 ± 3,518 RLU, respectively, when they were kept in DMEM for 24 h without amikacin, which was comparable to the luminescence intensity when these transformants were cultured in BHI broth (Fig. 1A). *R. equi* strains harboring virulence plasmids have been reported to cause necrotic death of macrophages and were released from the cells (24), decreasing the luminescence intensity at 48-h postinfection indicating that the extracellular bacteria (i.e., bacteria that could not be removed during washing at the time of infection or those released from the cells after macrophage growth and destruction) were killed by the antibiotic in the culture medium. In other words, the decrease in signals detected by the plate reader after 48 h of incubation was able to accurately quantify the proliferative potential of *R. equi* in the entire cultured cells, not just a part of the wells, and the problem with the fluorescent detection system might be solved.

When considering the application to experiments to evaluate the growth and pathogenicity of *R. equi* using *in vivo* imaging systems using laboratory animals, the detection of luminescent bacteria at around 37°C is necessary. However, when the Lux gene was expressed using P*_aphII_*, the luminescence intensity tended to be higher at 30°C incubation than at 37°C incubation in the culture medium adjusted to pH 6.5 (Fig. 1B). Therefore, using RNA-Seq to search for the expression levels exhaustively, we searched for the endogenous- and high-expression promoter of *R. equi* with little change under culture conditions based on the fluctuation values between neighboring genes. The TPM value of the KAREA_29370 gene (DUF3040 domain-containing protein; putative membrane protein) is 607-fold higher than that of its upstream KAREA_29380 gene. The TPM value of the KAREA_41760 gene (3-ketosteroid-9-alpha-hydroxylase subunit A) was 51-fold higher than that of its upstream KAREA_41750 gene, and that of the VapA gene was 10-fold higher than that of its upstream KAREA_49680 (Fig. 2A, B and C). In addition, the −10/−35 consensus sequence reported for *Streptomyces* (16) was conserved in the upstream sequences of both (Fig. 2D and E), suggesting that the upstream sequences of the above three genes may be endogenous high-expression promoter sequences. Three promoters (P*_29370_*, P*_41760_*, and P*_vapA_*) were linked to the *luxABCDE* operon and cloned into the pINT vector. All transformants showed luminescence (Fig. 3A), indicating that they can be used as autologous bioluminescence systems. In particular, the YSNR3, expressing the Lux gene by P*_29370_*, had almost the same luminescence intensity as YSNR1, expressing the Lux gene by P*_aphII_*, and the luminescence intensity of YSNR3 cultured at 37°C tended to be higher than that of YSNR1. In contrast, the YSNR5, expressing the Lux gene by P*_vapA_*, had significantly lower luminescence than YSNR1 and YSNR3 under all culture conditions. On the other hand, there was no significant difference in the number of viable bacteria among these transformants in each culture condition. The highest luminescence signal was observed at 37°C, pH 6.5 incubation. This result is consistent with the expression behavior of the VapA gene in the ATCC33701 strain (23, 25) and would be due to the same expression control mechanism via the promoter of the VapA gene. Next, we examined the proliferation of these transformants in cultured macrophages. The YSNR3, YSNR4, and YSNR5, which were ATCC33701-derived transformants, showed significantly higher luminescence values 24-h postinfection compared with the YSNR6, YSNR7, and YSNR8, which were ATCC33701_P–derived transformants, and its intensity decreased at 48 h (Fig. 3C). Similar results were obtained in the observation under the luminescence microscope (Fig. 3D). The YSNR5 showed the highest luminescence value in macrophages above the ATCC33701-derived transformants, and the luminescence intensity was similar or slightly higher than that of the YSNR1. Conversely, the luminescence intensity of the YSNR3, which showed the highest luminescence value when measured in cultured broth, was about half that of the YSNR5. It does not seem that there is a difference in the proliferation of each transformant because all the recipient strains are the same pVAPA-harboring ATCC33701. The difference in luminescence intensity is due to a difference in promoter activity, suggesting that P*_vapA_* may have functioned more sensitively to the environment in the macrophage phagolysosome (37°C, low pH).

Next, we examined the change in luminescence intensity by coexpression of the *luxABCDE* operon and the *frp* gene to increase the FMNH_2_ concentration in the *R. equi*. As shown in Fig. 4A, measurements of the broth cultured these *frp* coexpressed transformant (YSNR9, YSNR10, YSNR11, and YSNR12) showed no significant increase in luminescence intensity compared with the transformant without the *frp* (YSNR1, YSNR2, YSNR3, and YSNR6). However, the luminescence intensity in macrophages in YSNR9 and YSNR10 was enhanced up to 2-fold compared with that of YSNR1 and YSNR2 (without the *frp*). Similar results were also obtained in observations under the luminescence microscope (Fig. 4D). This might be because there was a limited amount of FMN, the substrate of FRP, in *R. equi* bacteria (or in the culture medium). Thus, FMNH_2_ could not be resynthesized beyond a certain amount. However, in the cultured cells, cell-derived FMN was supplied, FMNH_2_ was resynthesized, and the emission enhancement effect of the *frp* gene was observed.

Finally, we administered each transformant (YSNR9, YSNR10, YSNR11, and YSNR12) into the tail veins of the mice and verified if we could detect the growth of these transformants in the liver and spleen, where its growth was observed in previous reports (19). As expected, we detected luminescence in the liver and spleen of the pVAPA-harboring strains, including YSNR9 and YSNR11, and confirmed that the intensity and extent of the luminescence peaked on the third day after administration. However, in pVAPA-cured strains, including YSNR10 and YSNR12, there was no increase in luminescence signals (Fig. 5). This result indicates that the virulence plasmid-harboring autoluminescent *R. equi* can be detected within the liver and spleen with high sensitivity in the same mice. Based on our previous reports on the daily counts of ATCC33701 and ATCC33701_P- in organs (19), this autoluminescence system can detect signals only when the number of bacteria reaches 10^6^ to 10^7^ CFU/g of organ *in vivo* and not below 10^5^ CFU/g. This system will enable us to monitor bacterial proliferation over time in the same individual mice without killing them. Furthermore, it may be used to evaluate the pathogenicity of various strains (e.g., various wild strains or strains deleting genes involved in pathogenicity) and screen for preventive and therapeutic methods such as vaccines.

In conclusion, we produced an autologous bioluminescent strain of *R. equi* and established a method to evaluate its proliferation *in vitro* and *in vivo* quantitatively. Although this method needs further improvement, such as creating transformants that can maintain high-luminescence intensity regardless of environmental changes, this method can be used to not only evaluate the pathogenicity of various strains but also to screen preventive and therapeutic methods such as vaccines.

## MATERIALS AND METHODS

### Bacterial strains.

Bacterial strains used in this study are shown in Table 1. The *R. equi* ATCC33701, originally isolated from a pneumonic foal and the isogenic but avirulent *R. equi* ATCC33701_P-, a pVAPA-cured strain was used in this study (26). Escherichia coli strain HST08 Premium Competent Cells was purchased from TaKaRa Bio Inc. (Shiga, Japan). Vibrio parahaemolyticus strain NAHA-1 was kindly provided by Dr. Yukihiro Akeda (Research Institute for Microbial Diseases, Osaka University).

### Preparation of genomic DNA.

*R. equi* ATCC33701 and V. parahaemolyticus NAHA-1 were grown in BHI broth (Becton, Dickinson, Franklin Lakes, NJ, USA) for 16 h at 37°C with shaking (120 rpm). Genomic DNA (gDNA) was extracted using a QIAamp DNA minikit (Qiagen GmbH, Hilden, Germany) from the cultured cells. The concentration and purity of the extracted DNA were determined, respectively, using a Qubit dsDNA HS Assay Kits (Thermo Fisher Scientific, Waltham, MA, USA) and a NanoDrop OneC spectrophotometer (Thermo Fisher Scientific).

### Plasmid construction.

Tables 2 and 5 present plasmids and the primers used for plasmid construction. The luciferase expression vector (pMV306G13 + Lux), which carries *luxABCDE* operon and contains a Gram-positive enhanced translation signal in front of *luxA*, *luxC*, and *luxE*, was a gift from Brian Robertson & Siouxsie Wiles (27) (purchased from Addgene plasmid # 26160; http://n2t.net/addgene:26160; RRID: Addgene_26160). To generate an efficient vector series, we conducted vector manipulations. All PCRs were performed using Tks Gflex DNA polymerase (TaKaRa Bio Inc.) according to the manufacturer’s instructions. First, a DNA fragment of *luxABCDE* was amplified from pMV306G13 + Lux to partially overlap pINT::P*_aphII_*, using a primer set of No. 1 and No. 2 listed in Table 5, and another DNA fragment was prepared by *Mfe*I and NdeI (New England Biolabs, Ipswich, MA, USA) digestion of pINT::P*_aphII_*-egfp (10) and purification after agarose gel electrophoresis using a QIAquick Gel Extraction Kit (Qiagen GmbH, Hilden, Germany), resulting in DNA fragments removing *egfp* from pINT::P*_aphII_*-egfp. The two DNA fragments were then ligated with NEBuilder HiFi DNA Assembly Master Mix (New England Biolabs) and directly transformed into HST08 to generate pINT::P*_aphII_*-luxABCDE (pYSN1). Next, we replaced P*_aphII_* with another endogenous promoter of *R. equi*. A DNA fragment of endogenous promoters (P*_29370_*, P*_41760_*, or P*_vapA_*) was amplified from ATCC33701 gDNA, using each primer set of No. 3 and No. 4 for amplifying P*_29370_*, No. 5 and No. 6 for amplifying P*_41760_*, or No. 7 and No. 8 for amplifying P*_vapA_* listed in Table 5. Other DNA fragments from the pINT::P*_aphII_*-luxABCDE were amplified to remove the P*_aphII_* sequence and partially overlap each promoter sequence, using primers No. 9 and No. 10 for P*_29370_*, No. 11 and No. 12 for P*_41760_*, and No. 13 and No. 14 for P*_vapA_* listed in Table 5. The two DNA fragments were then ligated with NEBuilder HiFi DNA Assembly Master Mix and directly transformed into HST08 to generate pINT::P*_29370_*-luxABCDE (pYSN2), pINT::P*_41760_*-luxABCDE (pYSN3), and pINT::P*_vapA_*-luxABCDE (pYSN4), respectively. Finally, we added *frp* to pYSN1 and pYSN2. The first DNA fragments (*frp*) were amplified from NAHA-1 gDNA using each primer set of No. 15 and No. 16 for constructing pINT::P*_aphII_*-luxABCDE-P*_aphII_*-frp or No. 16 and No. 17 for constructing pINT::P*_29370_*-luxABCDE-P*_29370_*-frp. The second DNA fragments (P*_aphII_* or P*_29370_*) were amplified from pYSN1 or pYSN2 using each primer set of No. 18 and No. 19 or No. 20 and No. 21. The third DNA fragments (vector sequence) from the pYSN1 or pYSN2 were amplified using primers No. 22 and No. 23 for constructing pINT::P*_aphII_*-luxABCDE-P*_aphII_*-frp or No. 22 and No. 24 for constructing pINT::P*_29370_*-luxABCDE-P*_29370_*-frp listed in Table 5. Each primer was designed to overlap the appropriate number of bases. The three DNA fragments were then ligated with NEBuilder HiFi DNA Assembly Master Mix and directly transformed into HST08 to generate pINT::P*_aphII_*-luxABCDE-P*_aphII_*-frp (pYSN5) and pINT::P*_29370_*-luxABCDE-P*_29370_*-frp (pYSN6).

**TABLE 5:** Primers used in this study

No.	Name	Sequence (5′→3′)
1	pINT_PaphII_LuxA-E_IF_F	AGGATGAGGATCGTTTCATATGAAATTTGGAAACTTTTTG
2	pINT_PaphII_LuxA-E_IF_R	CGCTCTAGAACTAGTCAATTTCAACTATCAAACGCTTCGG
3	P29370_F_pINT_LuxA-E_delPaphII	GGGCCGATTCGCCGTGCTGGCCGCGAGCGA
4	P29370_R_pINT_LuxA-E_delPaphII	CAAATTTCATGGTACCTCCCCCGGCACTAAGAGTTGGC
5	P41760_F_pINT_LuxA-E_delPaphII	GGGCCGATTCGCCCGCCTGCACGGCCGCGG
6	P41760_R_pINT_LuxA-E_delPaphII	CAAATTTCATGTGTTCCTCCGTGCAGTGAGGGGTCGCCG
7	PvapA_F_pINT_LuxA-E_delPaphII	GGGCCGATTCGCGAGCCTGTGCCAGACCAA
8	PvapA_R_pINT_LuxA-E_delPaphII	CAAATTTCATCTTACTTCTCCTTTCGGACGTCGC
9	pINT_LuxA-E_noPaphII_P29370_F	GGGAGGTACCATGAAATTTGGAAACTTTTTGCTTACATACCAACCTCCCC
10	pINT_LuxA-E_noPaphII_P29370_R	CCAGCACGGCGAATCGGCCCCGGAGGACGC
11	pINT_LuxA-E_noPaphII_P41760_F	GGAGGAACACATGAAATTTGGAAACTTTTTGCTTACATACCAACCTCCCC
12	pINT_LuxA-E_noPaphII_P41760_R	GCAGGCGGGCGAATCGGCCCCGGAGGACGC
13	pINT_LuxA-E_noPaphII_PvapA_F	GAGAAGTAAGATGAAATTTGGAAACTTTTTGCTTACATACCAACCTCCCC
14	pINT_LuxA-E_noPaphII_PvapA_R	ACAGGCTCGCGAATCGGCCCCGGAGGACGC
15	frp_F_PaphII	ATCGTTTCATATGAACAGTACGATCCAAAC
16	frp_R_PaphII/P29370	CAAAAGCTGGTCACTTCTTTGCTAAGCC
17	frp_F_P29370	GGGAGGTACCATGAACAGTACGATCCAAAC
18	PaphII_F_frp	CGGTGGAGCTGGATCCGCACCGGCCCCG
19	PaphII_R_frp	TACTGTTCATATGAAACGATCCTCATCCTGTCTCTTGATCAGATCTTGATCC
20	P29370_F_frp	CGGTGGAGCTGCCGTGCTGGCCGCGAGC
21	P29370_R_frp	TACTGTTCATGGTACCTCCCCCGGCACTAAGAGTTG
22	pINT_PaphII/P29370_F_SacIsite	AAAGAAGTGACCAGCTTTTGTTCCCTTTAGTGAGGGTTAATTGCG
23	pINT_PaphII_R_SacIsite	GTGCGGATCCAGCTCCACCGCGGTGGCG
24	pINT_P29370_R_SacIsite	CCAGCACGGCAGCTCCACCGCGGTGGCG

### Transformation into *R. equi* and measurement of luminescence intensity.

Transformation of the above-constructed plasmid into ATCC33701 or ATCC33701_P- was performed following our previously described method (3). Briefly, 1 μg of each plasmid was electroporated into ATCC33701 or ATCC33701_P-. Transformants were recovered on LB agar containing 60 μg/mL apramycin, generating YSNR1-YSNR12, respectively. In all experiments, each transformant was precultured using BHI broth, and when OD = 0.05 was reached, 1/50 volume of each growth-broth was inoculated in BHI broth or conditioned broth (20 g/L peptone, 8 g/L NaCl, and 300 mL/L horse meat extract) adjusted to pH 6.5 or 8.0 with 1 M Tris–HCl buffer at 30°C or 37°C with shaking (120 rpm). At any given time, the cultured broth was collected and 100 μL was placed in SPL 96-well white plate (SPL Life Sciences, Korea), and the luminescence intensity was measured using an Infinite F200 PRO (Tecan, Mannedorf, Switzerland). In addition, 100 μL of the cultured broth was smeared on conditioned agar (10 g/L peptone, 5 g/L NaCl, 1g/L NaHCO_3_, Bacto agar 15g/L, and 300 mL/L horse meat extract) adjusted to pH 7.2, and the number of bacteria was measured.

### Intramacrophage growth of luciferase-expressing *R. equi*.

The murine macrophage-like cell line J774A.1 was seeded into CELLSTAR 96-well plates white with a transparent bottom (Greiner Bio-One International GmbH, Kremsmünster, Austria) at 4.0 × 10^4^ cells per well and cultured for 24 h in DMEM (Thermo Fisher Scientific) containing 10% fetal bovine serum (FBS, Corning, NY, USA) in a humidified 5% CO_2_ at 37°C. Cells were infected with each luciferase-expressing strain at a multiplicity of infection of 10. After incubation for 1 h at 37°C, the cells were washed three times with PBS(−) to remove extracellular bacteria. Then, 1 mL of DMEM containing 20, 2, or 0 μg/mL amikacin was added, and the cells were cultured for any given time in humidified 5% CO_2_ at 37°C. To measure the number of intracellular bacteria, the J774A.1 cells are disrupted with distilled water and pipetted slowly to disperse the bacteria to some extent. This solution (100 μL) was smeared on conditioned agar. The luminescence intensity was measured using an Infinite F200 PRO.

### Observation of infected *R. equi* in cells by luminescence microscopy.

The J774A.1 was seeded into a multiwell glass-bottom dish (D141400, Matsunami Glass Ind., Ltd., Osaka, Japan) at 8.0 × 10^4^ cells per well and cultured for 24 h in DMEM containing 10% FBS in a humidified 5% CO_2_ at 37°C. Infection of *R. equi* and cell culture were performed under the same conditions as above. The luminescence signals in the cells were detected using an IX83 microscope (Olympus, Tokyo, Japan).

### Whole-genome sequencing of ATCC33701 using Illumina MiSeq and Oxford Nanopore MinION.

Using ATCC33701 gDNA, the complete genome sequence was obtained by combining sequencing data from both Illumina MiSeq (Illumina, San Diego, CA, USA) and MinION (Oxford Nanopore Technologies, Oxford, UK) sequencers. Illumina sequencing was performed according to the manufacturer’s instructions. Briefly, an index-tagged library was prepared using the Nextera XT DNA Library Preparation Kit (Illumina), and 300-bp paired-end reads were sequenced on an Illumina MiSeq instrument. Nanopore sequencing was also performed according to the manufacturer’s instructions. Briefly, a DNA library was prepared using a Ligation Sequencing Kit and a Native Barcoding Expansion 1–12 (Oxford Nanopore Technologies). The prepared library was subsequently loaded into a MinION flow cell (R9.4.1; Oxford Nanopore Technologies). The MinION sequencing run was performed over 48 h. Base-calling and barcoding were performed using Guppy v2.3.7 (Oxford Nanopore Technologies). Hybrid assembly of the MiSeq and MinION reads was performed using Unicycler v0.4.2 (28). Complete genomes were annotated using DFAST (https://dfast.nig.ac.jp/).

### Total RNA extraction and RNA-Seq.

Total RNA was extracted from ATCC33701 cultured in BHI broth adjusted to pH 6.5 with 1 M Tris–HCl buffer for 48 h at 37°C with shaking (120 rpm). The cells were harvested by centrifugation at 5,000 × *g* for 10 min, and the total RNA was then extracted using a RNeasy minikit (Qiagen) according to the manufacturer’s instructions. The quantity of total RNA was measured using the Qubit RNA HS Assay Kits (Thermo Fisher Scientific). The quality of the total RNA was confirmed by calculating the A260/A280 absorbance ratio and performing gel electrophoresis using the Agilent 2200 TapeStation System. No degradation of 23S or 16S rRNA was observed. Total RNA (1 μg) was subjected to rRNA depletion using the Ribo-Zero rRNA Removal Kit (Bacteria) (Illumina) according to the manufacturer’s protocol. Following rRNA depletion, 10 ng of purified RNA was used for RNA-Seq library construction, which was prepared using a ScriptSeq Complete Kit (Bacteria) (Illumina) and FailSafe Enzyme Mix (Illumina). Whole transcriptome RNA-Seq was performed on an Illumina MiSeq instrument with 75-bp paired-end reads according to the manufacturer’s instructions. Gene expression levels were normalized as TPM reads via the “RNA-Seq analysis” mode of the CLC genomics workbench (Qiagen). The −10/−35 sequences within the upstream sequences of KAREA_29370 and KAREA_41760 genes were searched using PromoterHunter (29). The −10/−35 consensus sequence reported for *Streptomyces* sp. (16) was used as a matrix.

### *In vivo* bioluminescent imaging.

Slc:ddY mice were purchased from Japan SLC, Inc. (Shizuoka, Japan). They were housed at 22°C to 25°C under a 12/12 h light/dark cycle. The animal experiments were approved by the Animal Research Ethics Committees of Kitasato University School of Veterinary Medicine (permit number: 21-030) and conducted following the guidelines for animal experimentation. The strains YSNR9, YSNR10, YSNR11, and YSNR12 were cultured for 48 h at 30°C and adjusted to 2.0 × 10^6^ CFU per 200 μL, which is the inoculation volume. The cultured bacterial strains were administered to each mouse (three animals per condition) via the tail vein (19). Additionally, the infected mice were housed for 7 days and luminescence signals emitted from each transformant were imaged at 1-day intervals using an IVIS200 imaging system (Xenogen/PerkinElmer, MA, USA) with a 5-min exposure time. The total photons emitted were acquired using the Living Image software package. Mice were anesthetized in chambers containing 1.5% isoflurane inhalant (Pfizer, Tokyo, Japan).

### Statistical analysis.

Statistical analyses were performed using Statcel4 software (The Publisher OMS Ltd., Saitama, Japan). The correlation coefficient is calculated using Pearson’s correlation coefficient test at two variable values (i.e., RLU and CFU). The correlation coefficient can be calculated using the following formula;

$$R\left(X,Y\right)=\frac{\sum (x-\bar{x})(y-\bar{y})}{\sqrt{\sum {\left(x-\bar{x}\right)}^{2}\sum^{}{\left(y-\bar{y}\right)}^{2}}}$$

where $\bar{x}$ and $\bar{y}$ are the sample means AVERAGE(RLU) and AVERAGE(CFU). The data of luminescence intensity were analyzed initially by analysis of variance and then the TukeyKramer multiple comparisons. *P* values of <0.05 were considered significant.

### Data availability.

The accession numbers are as follows: AP025268 (ATCC33701 chromosome) and AP025269 (pVAPA_33701). Raw data from each experiment are included in the supplemental data set.
