# Supplementary material for: An Autobioluminescent Method for Evaluating In Vitro and In Vivo Growth of Rhodococcus equi
Source: Microbiol Spectr. 2022 May 31;10(3):e00758-22. doi: 10.1128/spectrum.00758-22 (PMC9241598; doi:10.1128/spectrum.00758-22)
Supplement: SUPPLEMENTAL FILE 1 — Supplemental material. Download spectrum.00758-22-s0001.pdf, PDF file, 0.2 MB [file spectrum.00758-22-s0001.pdf]

Supporting Figure

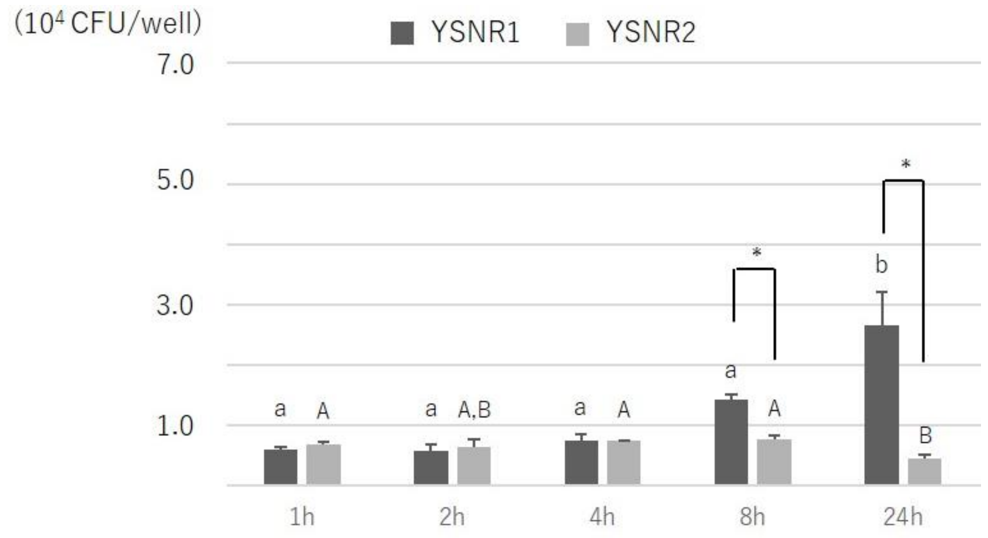

Fig S1. Comparison of the viability of YSNR1 (pVAPA-harboring transformant) and YSNR2 (pVAPA-cured transformant) after intracellular infection of J774A.1 macrophages. The number of colonies of YSNR2 after 8 h was significantly lower than that of YSNR1, and the number of colonies of YSNR2 decreased significantly with time, indicating that the bacteria did not grow and died. Experiments were performed in triplicate, and data are presented as the mean value ± standard deviation. Statistical analysis of changes in bacterial counts over time in the same strains was performed using ANOVA followed by the Tukey–Kramer multiple comparison test and the different letters indicate significant differences in the data ($p < 0.05$). On the other hand, statistical analysis in comparing the number of bacteria of each strain at the same time was performed using Student's t-test. Data labeled connected by asterisks indicate significant differences ($p < 0.05$).

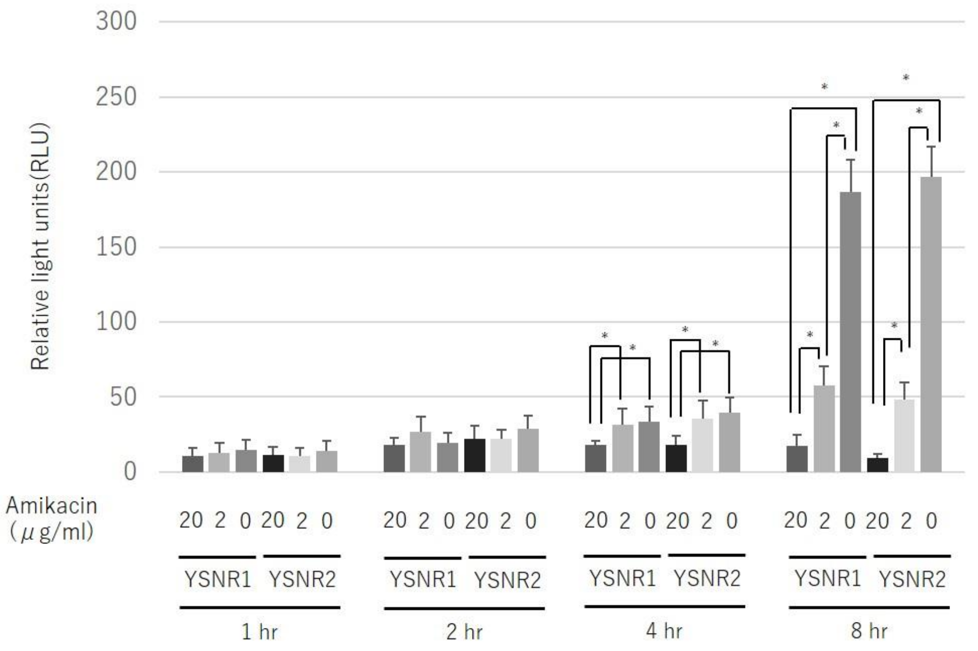

Fig. S2. Comparison of luminescence intensities with or without antibiotics in the culture medium in YSNR1 and YSNR2. After 4 h of incubation, the luminescence value significantly increased as the amikacin concentration decreased. Experiments were performed in septuplicate, and the data are presented as the mean value + standard deviation. Data labeled connected by asterisks indicate significant differences ($p < 0.05$).
